# Comparison of half-dose and full-dose triple therapy regimens for Helicobacter pylori eradication in patients with end-stage renal disease

**Published:** 2014

**Authors:** Mohammad Javad Ehsani Ardakani, Mohammad Aghajanian, Amir Ahmad Nasiri, Hamid Mohaghegh-Shalmani, Homayoun Zojaji, Iradj Maleki

**Affiliations:** 1 Imam Hossein Hospital, Shahid Beheshti University of Medical sciences, Tehran, Iran; 2 Gastroenterology and Liver Diseases Research Center, Shahid Beheshti University of Medical Sciences, Tehran, Iran; 3 Mazandaran University of Medical sciences, Sari, Iran

**Keywords:** Helicobacter pylori, Triple therapy regimens, Dictation

## Abstract

**Aim**: The aim of this study was to compare the half-dose and full-dose triple therapy regimens for *Helicobacter pylori *(*Hp*) eradication in patients with end-stage renal disease.

**Background**: *H. Pylori *is one the most important causes of dyspepsia in patients with end-stage renal disease (ESRD).

**Patients and methods**: Sixty-six patients with ESRD were enrolled in the study with *Hp *infection and peptic disease with a need of *Hp *eradication. Patients were randomly assigned to full-dose (A=35 patients) or half-dose group (B=31 patients). Patients received clarithromycin 500 mg, amoxicillin 1000 mg and omeprazole 20 mg twice daily in group A and clarithromycin 250 mg and amoxicillin 500 mg twice daily and omeprazole 20 mg once daily in group B for two weeks. Patients provided stool samples 4 weeks of completing study to assess the success of *Hp *eradication by *Hp*-specific stool antigen. Finally, the rate of eradication and complications were compared between two groups.

**Results**: The successful *Hp *eradication was achieved in 26 patients (74%) in group A and in 22 patients (74%) in group B. The difference between 2 groups was not statistically significant (p=0.973) (per protocol analysis).

**Conclusion**: Half-dose triple-therapy with clarithromycin, amoxicillin and omeprazole is as effective as full-dose triple-therapy to eradicate the *Hp *in patients with ESRD. According to lower toxicity level, complications and cost in half-dose regimen in this subset of patients, this protocol is advised.

## Introduction

*Helicobacter pylori *(*Hp*) is one of the most important causes of dyspepsia in patients with end-stage renal disease (ESRD). In renal failure patients, it is desirable to decrease the dosage of antibiotics used for *Hp *eradication (1). Most patients with end stage renal disease do not tolerate the usual dose of antibiotics for the eradication of *Hp *(2,3). 

Various antibiotics, such as metronidazole, clarithromycin, tetracycline, and amoxicillin, have been used in a wide variety of combinations for this purpose (4-6). Because of the development of resistant strains to various types of *Hp*, the high incidence of side effects, complicated regimens, and poor compliance, the desirable treatment has yet to be established (7,8).

The American College of Gastroenterology has recommended that all infected patients with *Hp *causative ulcer should eradicate their infection (9). Some studies suggested half-dose triple therapy for 10–14 days is effective as full dose triple therapy (11, 12).

Patients with end-stage renal disease frequently have complications of the upper gastrointestinal tract. However, the efficacy of this short-course triple therapy has not been well documented in these patients (10). 

A common regimen rely on metronidazole includes clarithromycin, amoxicillin, and either omeprazole or dictation (11). However, the rate of clarithromycin resistance is 7–15% in European countries as well as US (11-14). The prevalence of *Hp *in end-stage renal disease patients is the same in individuals with normal renal function (15-17). 

The main purpose of current study was to compare the success rate of *Hp *eradication in patients with ESRD on hemodialysis between half-dose and full-dose triple antibiotic therapy. Any dose reduction in antibiotics prescribed in these patients will ensure compliance and hence the success rate of the regimen. 

## Patients and Methods

This prospective, double-blinded clinical trial was conducted between March 2011 and October 2011. Sixty-six patients aged 19–79 years with ESRD were enrolled in the study with *Hp *infection and peptic disease with a need of *Hp *eradication. They underwent dialysis at different referral centers in the Tehran city, Iran. The mean duration of dialysis was 39.43±29.7 months.

Informed consent was given to patients before their initial evaluation for upper GI endoscopy. Those who were diagnosed with *H. pylori* on initial testing were included in the study. To confirm *H. pylori* infection, two types of tests were used: *H. pylori* stool Ag (DRG® *Helicobacter Pylori* Ag (stool) ELISA, USA), and the urease breath test (UBT, based on the modified European protocol). If one of these tests were positive, the patients were considered infected by *H. pylori*.

Subsequently, patients were randomly assigned to one of two treatments groups including full-dose group (A=35 patients) or half-dose (B=31 patients) for two weeks of triple therapy. Patients received clarithromycin 500 mg, amoxicillin 1000 mg and omeprazole 20 mg twice daily in group A and clarithromycin 250 mg and amoxicillin 500 mg twice daily and omeprazole 20 mg once daily in group B for 2 weeks. 

Four weeks after completing therapy, the stool antigen and UB tests were performed to assess the success of *Hp *eradication by *Hp*-specific stool antigen. Finally, the rate of eradication and complications were compared between 2 groups.

The exclusion criteria included a history of previous *H. pylori *treatment and any antibiotics customs over the previous 5 months and recorded allergy to any usage of medications. Any side effects and symptom changes were recorded. Patients’ compliance was evaluated at the end of treatment. The Shahid Beheshti University of Medical Sciences ethics committee approved the study. 

**Statistical analysis**

A Chi-square test with Pearson’s correction was performed to compare string variables between groups. A student’s independent T-test for quantitative variables with normal distribution and a Mann-Whitney U-test for numeric variables without normal distribution were conducted. The paired t-test was used to compare variables before and after *H. pylori*. P-values of less than 0.05 were considered significant. 

## Results

Out of the 95 end-stage renal disease patients referred to the dialysis centers of the Shahid Beheshti University hospitals, Tehran, 66 patients with a history of dyspepsia and positive results of *Hp* diagnostic tests were enrolled into this study. Twenty-one patients (60%) in group A and 17 patients (54.8%) in group B were female, while 14 patients (40%) in group A and 14 patients (45.2%) in group B were male. The mean age of patients in the two groups was 43 years. Demographic features of the patients in the two groups are shown in Table 1.

**Table 1:** Comparison of the demographic and endoscopy variables in the two groups

	Group A	Group B	P value
	N=35 (%)	N=31 (%)	
Age	46.2±14.1	49.0±17.2	N.S.^*^
Female	21(60)	17(54.8)	N.S.
Smoker	16 (45.7)	14(45.1)	N.S.
Duodenal ulcer & erosions	26(74)	21 (74	N.S.
Gastric Ulcer & erosions	9 (25.7)	8 (25.8)	N.S.

* Not Significant

Biochemistry tests performed to measure the serum creatinine, blood urea nitrogen, albumin, triglyceride, cholesterol, calcium, and phosphorous. As shown in Table 2, only the mean creatinine level in group A was significantly higher than in group B after triple therapy (P<0.05) and for the rest of variables no statistical differences were found between the two groups. All patients had completely taken prescribed regimen.

The successful *Hp *eradication was achieved in 26 patients (74%) in group A (full-dose) and 22 patients (74%) in group B (half-dose). The difference between 2 groups was not statistically significant (p=0.973) (per protocol analysis). 

Table 3showed the prevalence of side effects in study groups. The rate of bitter taste of the mouth and abdominal distention were significantly higher in group A (73.6% and 39.7%, respectively) compared to group B (41.2% and 18.3%, respectively) (p= 0.014 and 0.04, respectively). The rate of other complications did not differ between the groups. There are no statistical significant differences between two groups in terms of gender, age, and duration of dialysis. No one quit the regimen due to drug side effects. 

**Table 2:** H. pylori eradication efficacy of triple therapy in hemodialysis patient’s tests\groups

^*^Variables/Groups	Group A	Group B	P value
	N=35 (%)	N=31 (%)	
Cr, mg/dL	7.9	10.3	0.04
BUN, mg/dL	66.5	67.4	0.71
Alb, mg/dL	4.7±0.49	4.9±0.53	0.45
TG, mg/dL	137±72	147±84	0.64
Chol, mg/dL	160±39	154±29	0.79
Hgb, g/dL	10.4±1.5	10.8±1.8	0.56
Ca, mg/dL	9.2±1	9.1±0.7	0.61
P, mg/dL	5.9±1.9	5.8±1.3	0.88
BMI, kg/m2	24.7±3.7	25.6±4.2	0.49

^*^Cr: creatinine; BUN: blood urea nitrogen; Alb: serum albumin; TG: triglyceride; Chol, cholesterol; Hgb: hemoglobin; Ca: calcium; P: phosphore; BMI, body mass index

**Table 3:** The prevalence of gastrointestinal symptoms in studies groups

	Group A	Group B	P Value
	N=35(%)	N=31(%)	
Bitter mouth	30(85.7)	11(35.4)	0.014
Abdominalpain	11(31.4)	8(25.8)	N.S.^*^
Nausea/vomit	4(11.4)	3(9.6)	N.S.
Diarrhea	4(11.4)	3(9.6)	N.S.
Bloating	16(45.)	3(9.6)	N.S.
Pruritus	2(5.7)	3(9.6)	N.S.
Headache	7(20)	3(9.6)	N.S.
Insomnia	2(57)	0(0)	N.S.
Skin rash	0(0)	0(0)	N.S.

* Not Significant

## Discussion

*Helicobacter pylori *is a common human pathogen and is involved in the development of chronic gastritis, peptic ulcer disease, primary gastric mucosa-associated lymphoid tissue (MALT) lymphoma, and gastric cancer in more than 90% of cases (18).

Different gastrointestinal symptoms such as abdominal pain, functional dyspepsia, early fullness, bloating, nausea and vomiting and belching are common in hemodialysis patients (4). Hemorrhage in digestive tract is sometimes critical in dialysis patients. Moreover, peptic ulcer disease is diagnosed in up to 25% of patients with end-stage renal disease (10, 19).

Various reports have shown that multiple drug regimens are beneficial for *H. pylori* eradication (2,9,11,12). For this purpose, different antibiotics regimens using metronidazole, clarithromycin, azithromycin, tetracycline, amoxicicilin, furazolidone, levofloxacin, and rifabutin have been introduced. 

We performed a randomized, blinded study compared two regimens of full dose and half-dose 14-day triple therapy for *H. pylori* eradication in patients with end-stage renal disease, who were on hemodialysis. We achieved a high rate of *H. pylori* eradication (73%) in both groups, with no statistical differences between the two regimens (p=0.973).

Similar to our study, Vcev et al*. *found that eradication rates of pantoprazole and amoxicillin with either azithromycin or clarithromycin was 71% and 81%, respectively (20). 

Our study showed that after triple therapy for* H. pylori *eradication in both groups, the mean creatinine level in group with full doses was significantly higher than in group with half dose. Study by Furuta et al. in 2002 showed that the non-uremic *H. pylori *infected group had a significantly greater frequency of hypo-proteinemia as compared with the *H. pylori*-negative group (21). This finding is incompatible to our study.

Although proton-pump inhibitors have antimicrobial activity against *H. pylori, *the outcomes of monotherapy with a proton-pump inhibitor have been disappointing. The result of this study indicated that half dose triple-therapy with clarithromycin, amoxicillin and omeprazole is as effective as full-dose triple-therapy to eradicate the *Hp *in patients with ESRD. According to lower toxicity, complications and cost of half-dose regimen in this subset of patients, this protocol is advised. 

However, one should remember that the achieved eradication rates were not acceptable in eradication of *Hp*. Moreover, this problem has been seen in previous trials on ESRD subjects. To solve this issue, other trials with larger numbers of subject and newer eradication regimens are recommended.
